# Necrosis of Lower Jaw

**Published:** 1863-11

**Authors:** 


Necrosis of Lower Jaw.—Dr. Voss presented two sides of the lower jaw, together with several smaller pieces, which he had removed from a child seven years of age, on account of necrosis. The Doctor saw the child for the first time in October last, and found the jaw very much swollen, and there was also a fœtid discharge from some fistulous openings in the mouth. By introducing a probe into these openings, the nature of the disease was readily discovered. Previous to the attack of periostitis, which by the way, occurred simultaneously on the two sides, the child had never suffered from any sickness. The dead portions of bone were removed from the inside by removing the fistulæ, and consisted of the two articular processes, coronoid processes, angle, and that portion of the body of the bone not included in the chin. Subsequently to this, various other pieces of bone were removed, including several of the teeth. The patient made a good recoveiy, the wounds healed up kindly, and new bone has appeared in place of that which has been removed. There is no deformity perceptible, save a retraction of the chin and a want of prominence at either angle of the jaw. In consequence of this retraction, the tongue is thrown back somewhat upon the larynx, and the child has a slightly noisy respiration. It was the intention of Dr. Voss to show the child in connection with the specimens, but the inclemency of the weather prevented.

Dr. Elliot remarked, that in a case where Dr. Carnochan removed the entire lower jaw, the deformity was not enough to be noticed by any save a professional person.

Dr. Garrish referred to the case of a child, five years of age, who lost three-fourths of the lower jaw by necrosis, the result of salivation from only four grains of calomel given at a dose.

Dr. Voss stated that there was no evidences of either phosphorus or mercury acting as a cause of the disease; in fact, he was at a loss to decide what was the cause; unless, perhaps, the second dentition might have had something to do with it. It was certainly very strange to him how both sides of the jaw were simultaneously affected.—*American Med. Times.*

